# Reading tree leaves: inferring speciation and extinction processes using phylogenies

**DOI:** 10.1098/rstb.2023.0309

**Published:** 2025-02-20

**Authors:** Bruce Rannala, Ziheng Yang

**Affiliations:** ^1^ Department of Evolution and Ecology, University of California, Davis, CA 95616, USA; ^2^ Department of Genetics, Evolution, and Environment, University College London, London WC1E 6BT, UK

**Keywords:** Yule process, generalized birth–death process, identifiability, statistical inference, phylogenetic trees

## Abstract

The birth–death process (BDP) is widely used in evolutionary biology as a model for generating phylogenetic trees of species. The generalized birth–death process (GBDP) allows rate variation over time, with speciation and extinction rates to be arbitrary functions of time. Here we review the probability theory underpinning the GBDP as a model of cladogenesis and recent findings concerning its identifiability. The GBDP with arbitrary continuous rate functions has been shown to be non-identifiable from lineage-through-time data: even with species phylogenies of infinite size the parameters cannot be estimated. However, a restricted class of BDPs with piecewise-constant rates has been shown to be identifiable. We review and illustrate these results using simple examples and discuss their implications for biologists interested in inferring the past tempo and mode of evolution using reconstructed phylogenetic trees.

This article is part of the theme issue ‘“A mathematical theory of evolution”: phylogenetic models dating back 100 years’.

## 1. Introduction

Phylogenetic relationships among species are a consequence of complex historical processes, including population fragmentation and divergence, genetic drift and ecological adaptation, ultimately leading to increased genetic isolation and generating new species. These processes naturally lead to hierarchical relationships among extant and extinct species, including shared ancestral species existing at various times in the past. An evolutionary tree representation of relationships among species has been used since the time of Darwin in the nineteenth century [1]. ‘Tree thinking’ has become one of the fundamental unifying principles of evolutionary biology [2].

**Figure 1.**
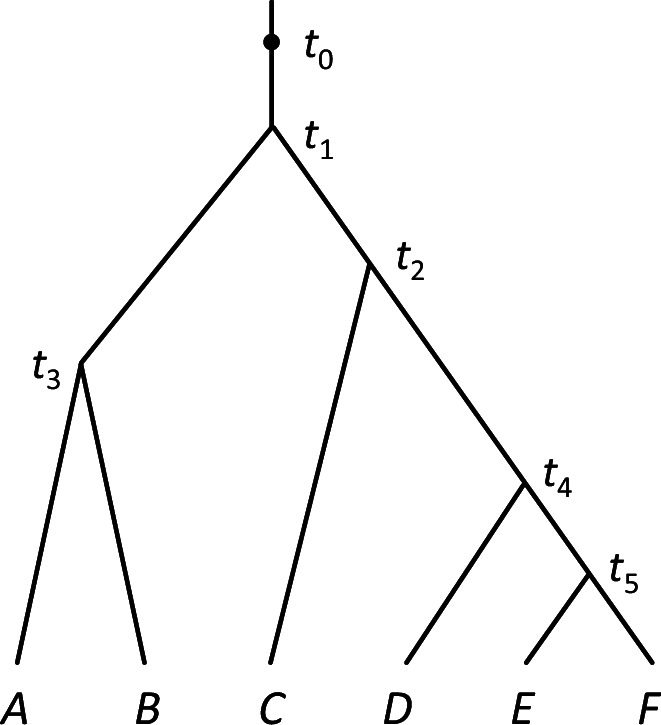
A species phylogeny for six species with internal nodes rank-ordered by age. A tree with labelled tips and rank-ordered internal nodes is called a ‘labelled history’ or ‘ranked rooted tree’. The time of origin, $t_{0}$, is used to formulate the birth–death process (BDP) model of cladogenesis. In models considered in this article, the tree topology is not informative about the parameters in the BDP, and only the speciation times (or equivalently the 'lineage through time' or LTT data) constitute the data: $t_{1},...,t_{5}$.

Relationships among species over time are predominantly represented as a binary tree (phylogeny) with branch lengths proportional to time (see figure 1). Although genomic datasets frequently contradict this tree view, supporting more nuanced species relationships defined by introgression and horizontal gene transfer which generates horizontal connections between branches and produces networks rather than binary phylogenetic trees [3], binary trees still provide a useful backbone onto which introgression may be superimposed. Phylogenetic trees also provide a record of the tempo and mode of past evolution in different groups of species [4].

Unique geological and environmental events are expected to play a major role in generating phylogenetic relationships among species. Any process-based mechanistic model of speciation and extinction will be highly complex and group-specific, omit important unobserved factors, and be unrealistic. Nonetheless, biologists have a deep interest in discovering the forces that generated biological diversity, such as geological upheavals, climatic catastrophes and extraterrestrial collisions that may be associated with radiative speciations or mass extinctions. Unfortunately, the extreme stochasticity of even ‘neutral’ branching processes with constant speciation and extinction rates can mislead inferences based solely on apparent diversification rates on the phylogenetic tree or based on ‘reading of the tree leaves’. To infer speciation and extinction processes rigorously requires a proper statistical inference framework.

To study speciation and extinction using estimated phylogenies a model of the processes generating the phylogeny is needed. Here we focus on a class of stochastic continuous-time birth–death processes (BDPs) whose study began a century ago with the pure-birth process proposed by Udny Yule [5]. BDPs have provided simple parametric models that allow formal statistical inference procedures to be used to estimate birth (speciation) and death (extinction) rates from phylogenetic trees. BDPs are obviously not an accurate model of the true underlying processes; however, they attempt to capture the most important features with per-species rates of speciation and extinction that may vary through time or across lineages. BDP-based inference methods have been widely used in evolutionary biology [6], but such approaches have also been criticized on several grounds [7–9]. Here we review some recent criticisms and controversies, particularly concerning whether certain BDPs can be distinguished by data. We provide a description of the most widely used BDP model that allows variable rates, the generalized birth–death process (GBDP) [10], and consider the statistical properties of inference methods under the model.

Two distinct approaches have been taken to extend BDPs to account for variable rates of speciation and extinction: variation of rates through time (with rates shared across all contemporary species) [11] and variation of rates across lineages [12,13]. An important difference is that in the first (rate variation through time), all tree topologies with rank-ordered internal nodes and labelled tips (so-called labelled histories; see §4 below) have equal probabilities. As a consequence, divergence times on the phylogeny or the so-called ‘lineages through time’ (LTT) summary [11,14] provide a sufficient statistic for estimating speciation and extinction rates. This is not the case in the second approach (with rate variation across lineages), in which tree topologies are also informative about the rates. In this article, we consider only models of rate variation through time. However, many of our observations are of relevance for inferences under the second class of models as well.

## 2. Phylogenetic trees as data

The data used to infer speciation and extinction rates are usually phylogenetic trees of species. Here we assume that fossils are excluded and all species tips on the tree are contemporaneous; this is typical of most analyses (we do not consider applications of BDPs as models of virus transmission with non-contemporaneous tips or ancient DNA samples). Such ‘time trees’ are generated by analysing sequence data from a sample of contemporary species using phylogenetic inference methods that assume a molecular clock or a ‘relaxed clock’ [15,16]. The branch lengths of the phylogeny are in units of the expected number of substitutions but can be converted to absolute geological time by use of fossil calibrations. An example of a phylogenetic tree is shown in figure 1. Such trees provide the history of surviving species; extinct species are effectively removed from the tree if they are not direct ancestors. Nee *et al*. [11] referred to this tree of relationships for only the contemporary species as the ‘reconstructed tree’.

Comparative studies using BDPs typically do not distinguish between gene trees and species trees although processes such as incomplete lineage sorting could lead to gene tree versus species tree conflicts and biased species divergence time estimates [17,18]. Also, treating inferred phylogenetic trees as observed data to infer speciation and extinction rates ignores phylogenetic uncertainty (in particular uncertainty in branch lengths). One solution to this problem is to integrate over the trees while estimating rates through Bayesian phylogenetic inference with a BDP used as a prior [19,20]. Another approximation uses trees sampled from a separate Bayesian phylogenetic analysis through Markov chain Monte Carlo (MCMC) and averages inferences over the MCMC sample [21]. Although potentially important, we ignore species tree versus gene tree conflicts and phylogenetic uncertainty in our considerations, focusing instead on the ‘best-case’ scenario by using a known phylogenetic (species) tree to infer rates.

## 3. The Yule process

The Yule process [5] is a continuous-time pure-birth process in which birth (speciation) occurs at the per-lineage rate *λ* and there are no extinctions. The process begins with at least one lineage and the number of lineages is strictly increasing. For calculating probabilities on phylogenetic trees the only transition probability needed is $p_{1}(t)$, the probability that a lineage existing at time 0 has exactly one descendant time t later. If the process is initiated with $n_{0}=1$ lineages at time t=0 the finite difference equation is


(3.1) $$\begin{array}{ccc} & p_{1}(t+\Delta t)=p_{1}(t)[1-\lambda \Delta t]+o(\Delta t), & \end{array}$$

and the limiting ordinary differential equation (ODE) is


(3.2) $$\begin{array}{rl} \lim_{\Delta t\to 0}\frac{p_{1}(t+\Delta t)-p_{1}(t)}{\Delta t} & =\lim_{\Delta t\to 0}\left(-p_{1}(t)\lambda +\frac{o(\Delta t)}{\Delta t}\right)\\ \frac{\;dp_{1}(t)}{\;dt} & =-p_{1}(t)\lambda . \end{array}$$

Solving (3.2) with initial condition $p_{1}(0)=1$ gives


(3.3) $$\begin{array}{ccc} & p_{1}(t)=e^{-\lambda t}. & \end{array}$$

The general formula for $p_{n}(t)$ can be obtained by iteratively solving the successive ODEs. For example,


(3.4) $$\begin{array}{ccc} & p_{2}(t+\Delta t)=p_{1}(t)\lambda \Delta t+p_{2}(t)(1-2\lambda \Delta t)+o(\Delta t), & \end{array}$$

which gives the ODE


(3.5) $$\begin{array}{ccc} & \frac{\;dp_{2}(t)}{\;dt}=-2p_{2}(t)\lambda +p_{1}(t)\lambda =-2p_{2}(t)\lambda +e^{-\lambda t}\lambda . & \end{array}$$

With the initial condition $p_{2}(0)=0$, this has the solution


(3.6) $$\begin{array}{ccc} & p_{2}(t)=e^{-2\lambda t}(e^{\lambda t}-1). & \end{array}$$

In general, $p_{n}(t)$ is given as


(3.7) $$\begin{array}{ccc} & p_{n}(t)=e^{-\lambda t}(1-e^{-\lambda t}{)}^{n-1}. & \end{array}$$

## 4. Probability of the reconstructed tree

Edwards [22] considered the calculation of the probability of a phylogenetic tree under a Yule process. The same approach applies under the GBDP considered later in this article with an obvious substitution of probability terms. We therefore review the results for the Yule process in some detail. Edwards refers to a phylogenetic tree with labelled tips and rank-ordered internal nodes as a ‘labelled history’ (figure 1). This is the fundamental tree structure under both the coalescent model and the birth–death model. Contribution [23] of this issue further defines the concept of a labelled history, extending it to multifurcating trees. Rank-ordering the heights (times) of speciation events on a tree with a stem at time $t_{0}$, we have $𝐭=\{t_{1},t_{2},\ldots ,t_{n-1}\}$, where $t_{1}$ is the height of the root node (figure 1). A speciation event occurs on any of the i−1 branches of the tree that exist during time interval $\left(t_{i}+\Delta t,t_{i}\right)$ with probability $(i-1)\lambda \Delta t_{i}$ and the resulting lineage leaves exactly one descendant at time present with probability $p_{1}(t_{i})$. The joint probability of having, after time $t_{0}$, n tips and node heights 𝐭 is then


(4.1) $$\begin{array}{ccc} & f(𝐭,n|t_{0})=p_{1}(t_{0})\prod_{i=1}^{n-1}i\lambda p_{1}(t_{i})=(n-1)!\lambda^{n-1}\prod_{i=0}^{n-1}p_{1}(t_{i})=(n-1)!\lambda^{n-1}e^{-\lambda \sum_{i=0}^{n-1}t_{i}}. & \end{array}$$

Integrating over the branch lengths for a fixed stem age gives the marginal probability of n descendants after time $t_{0}$,


(4.2) $$f(n|t_{0})=\int_{t_{1}=t_{0}}^{t_{2}}\cdots \int_{t_{n-1}=0}^{t_{n-2}}(n-1)!\lambda^{n-1}e^{-\lambda \sum_{i=0}^{n-1}t_{i}}\text{d}t=e^{-n\lambda t_{0}}(e^{\lambda t_{0}}-1{)}^{n-1}.$$

Some authors [24] use the probability density function (PDF) of speciation times conditional on having n tips and the stem age $t_{0}$ (i.e. time that BDP was initiated with a single lineage):


(4.3) $$\begin{array}{ccc} & f(𝐭|t_{0},n)=\frac{(n-1)!\lambda^{n-1}e^{-\lambda \sum_{i=0}^{n-1}t_{i}}}{p_{n}(t_{0})}=\frac{(n-1)!\lambda^{n-1}e^{-\lambda \sum_{i=0}^{n-1}t_{i}}}{e^{-n\lambda t_{0}}(e^{\lambda t_{0}}-1{)}^{n-1}}. & \end{array}$$

However, the stem age is not a property of observed phylogenetic trees so it must be dealt with, for example, by integrating it out using a Bayesian prior. Other authors [19] follow [22] and instead condition on the number of tips n and the age of the root (which is observed in the phylogenetic tree):


(4.4) $$Pr(n|t_{1})=\sum_{i=1}^{n-1}p_{i}(t_{1})p_{n-i}(t_{1})=(n-1)e^{-2\lambda t_{1}}(1-e^{-\lambda t_{1}}{)}^{n-2},$$

and thus


(4.5) $$\begin{array}{ccc} & f(𝐭|t_{1},n)=\frac{(n-1)!\lambda^{n-1}e^{-\lambda \sum_{i=1}^{n-1}t_{i}}}{\mathrm{Pr}(n|t_{1})}=\frac{(n-2)!\lambda^{n-1}e^{-\lambda \sum_{i=1}^{n-1}t_{i}}}{e^{-2\lambda t_{1}}(1-e^{-\lambda t_{1}}{)}^{n-2}}. & \end{array}$$

There may be situations where a stem age is a biologically meaningful concept (and a prior may be available). For example, in branching models of cell fate in an organism, the stem age is the age of the zygote. However, in phylogenetic systematics the meaning of a stem age is typically obscure. Conditioning on stem age, equation (4.3) can be rearranged to give


(4.6) $$f(t|t_{0},n)=\frac{p_{1}(t_{0})}{p_{n}(t_{0})}\prod_{i=1}^{n-1}i\lambda p_{1}(t_{i})=\frac{1}{{\left(1-e^{-\lambda t_{0}}\right)}^{n-1}}\prod_{i=1}^{n-1}i\lambda p_{1}(t_{i})=(n-1)!\prod_{i=1}^{n-1}\frac{\lambda p_{1}(t_{i})}{1-e^{-\lambda t_{0}}}=(n-1)!\prod_{i=1}^{n-1}\frac{\lambda e^{-\lambda t_{i}}}{1-e^{-\lambda t_{0}}}.$$

This is the joint density of the order statistics for n−1 independent and identically distributed (i.i.d.) random variables [22] with kernel density:


(4.7) $$f(x)=\frac{\lambda e^{-\lambda x}}{1-e^{-\lambda t_{0}}},\;for\;0\leq x\leq t_{0}.$$

In the case of the Yule process, the kernel density is a simple truncated exponential. This order-statistic form holds for the BDP as well [24] (see also [25]). This representation suggests an efficient method for simulating from a BDP conditional on n and $t_{0}$ [24], as used by [26] to simulate large trees under the BDP. In this procedure, n−1 i.i.d. variables are simulated from the kernel (eq. 9 of [24]) and rank ordered to obtain a sample from the joint density.

## 5. The generalized birth–death process

The Yule process described above has obvious limitations. Extinction is not allowed in the model, and the speciation rate is assumed constant. Feller proposed a linear BDP to incorporate extinction, with constant rates of speciation and extinction (λ,μ). Kendall [10] developed a ‘generalized’ birth–death process (GBDP) model in which the birth rate λ(t) and death rate μ(t) are deterministic continuous functions of time. Remarkably, this generalized process still allows exact analytical solutions. Explicit solutions are available for $p_{i}(t)$, the probability that i descendants exist for a single lineage after t units of time. Of particular interest for calculating the PDF of phylogenetic trees using Edwards’s [22] method are the probabilities $p_{1}(t)$ and $p_{0}(t)$ that exactly one descendant, or zero descendants, respectively, exist after time t. Note that the stochastic process runs forward in time but t measures time in the past with the present time being t=0. For example,


(5.1) $$p_{0}(t)=1-\frac{1}{1+\int_{0}^{t}e^{\int_{0}^{s}[\mu (y)-\lambda (y)]\text{d}y}\mu (s)\;\text{d}s}$$

is the probability that all descendants of a single lineage have died out by time t. Equation (5.1) assumes that all extant species descended from a particular ancestral node are sampled (i.e. complete sampling). This is the *lineage extinction probability* E(t) in [27] and Φ(t) in [28] (in the special case of complete sampling). We will instead use $p_{0}(t)\equiv \Phi (t)\equiv E(t)$, to be consistent with Kendall’s original notation.

Nee *et al*. [11] added random sampling to the linear BDP. If a fraction ρ of lineages are randomly sampled at present, the equivalent probability is determined by the finite difference equation


(5.2) $$\begin{array}{ccc} & p_{0}(t+\Delta t)=p_{0}(t)[1-\lambda (t+\Delta t)\Delta t-\mu (t+\Delta t)\Delta t]+\mu (t+\Delta t)\Delta t+p_{0}(t{)}^{2}\lambda (t+\Delta t)\Delta t+o(\Delta t). & \end{array}$$

The three terms represent the probabilities that (i) no events occurred during the interval (t+Δt,t), with probability [1−λ(t+Δt)Δt−μ(t+Δt)Δt], and the lineage subsequently went extinct, with probability $p_{0}(t)$; (ii) a death occurred, with probability μ(t+Δt)Δt, so that the lineage went extinct during (t+Δt,t); and (iii) a birth occurred, with probability λ(t+Δt)Δt, and both lineages subsequently went extinct, with probability $p_{0}(t{)}^{2}$. Taking limits, we obtain the ODE


(5.3) $$\begin{array}{rl} \lim_{\Delta t\to 0}\frac{p_{0}(t+\Delta t)-p_{0}(t)}{\Delta t} & =\lim_{\Delta t\to 0}\left(-p_{0}(t)[\lambda (t+\Delta t)+\mu (t+\Delta t)]+\mu (t+\Delta t)+p_{0}(t{)}^{2}\lambda (t+\Delta t)+\frac{o(\Delta t)}{\Delta t}\right),\\ \frac{\text{d}p_{0}(t)}{\text{d}t} & =-p_{0}(t)[\lambda (t)+\mu (t)]+\mu (t)+p_{0}(t{)}^{2}\lambda (t). \end{array}$$

Solving the equation with the initial condition $p_{0}(0)=1-\rho$ gives


(5.4) $$\begin{array}{ccc} & p_{0}(t)=1-\frac{e^{\int_{0}^{t}\lambda (s)-\mu (s)\;ds}}{\frac{1}{\rho }+\int_{0}^{t}\lambda (s)e^{\int_{0}^{s}\lambda (y)-\mu (y)dy}\;ds}. & \end{array}$$

For constant λ and μ equation (5.4) simplifies to


(5.5) $$\begin{array}{ccc} & p_{0}(t)=\frac{\rho \mu e^{(\lambda -\mu )t}+\lambda (1-\rho )-\mu }{\rho \lambda e^{(\lambda -\mu )t}+\lambda (1-\rho )-\mu }. & \end{array}$$

In the case of constant λ and μ with ρ=1, both equation 5.1 and equation 5.4 simplify to


(5.6) $$\begin{array}{ccc} & p_{0}(t)=\frac{\mu (e^{(\lambda -\mu )t}-1)}{\lambda e^{(\lambda -\mu )t}-\mu }. & \end{array}$$

In calculating the PDF for labelled histories we also need the probability that a lineage has a single descendant after time t, the solution of which (with complete sampling) from Kendall [10] is


(5.7) $$\begin{array}{ccc} & p_{1}(t)=[1-p_{0}(t)](1-\eta_{t}), & \end{array}$$

where


(5.8) $$\eta_{t}=1-\frac{1}{e^{-\int_{0}^{t}[\mu (s)-\lambda (s)]ds}\left[1+\int_{0}^{t}e^{\int_{0}^{s}[\mu (y)-\lambda (y)]dy}\mu (s)ds\right]}.$$

To obtain the PDF with sampling, a finite difference equation is again formulated


(5.9) $$\begin{array}{ccc} & p_{1}(t+\Delta t)=p_{1}(t)[1-\lambda (t+\Delta t)\Delta t-\mu (t+\Delta t)\Delta t]+2p_{1}(t)p_{0}(t)\lambda (t+\Delta t)\Delta t+o(\Delta t). & \end{array}$$

The two terms are the probabilities (i) that no events occur during the interval (t+Δt,t), with probability 1−λ(t+Δt)Δt−μ(t+Δt)Δt, and exactly one lineage survives after time t, $p_{1}(t)$; and (ii) that a birth occurs during (t+Δt,t), with probability λ(t+Δt)Δt, and exactly one of the lineages survives to present, with probability $2p_{1}(t)p_{0}(t)$. We have


(5.10) $$\begin{array}{rl} \lim_{\Delta t\to 0}\frac{p_{1}(t+\Delta t)-p_{1}(t)}{\Delta t} & =\lim_{\Delta t\to 0}[-p_{1}(t)[\lambda (t+\Delta t)+\mu (t+\Delta t)]+2p_{1}(t)p_{0}(t)\lambda (t+\Delta t)+\frac{o(\Delta t)}{\Delta t}],\\ \frac{\text{d}p_{1}(t)}{\text{d}t} & =-p_{1}(t)[\lambda (t)+\mu (t)]+2p_{1}(t)p_{0}(t)\lambda (t). \end{array}$$

This ODE is solved using the initial condition $p_{1}(0)=\rho$ to account for sampling, which gives


(5.11) $$\begin{array}{ccc} & p_{1}(t)=\frac{\rho \;\exp\{2\int_{0}^{t}\lambda (s)p_{0}(s)\;ds\}}{\;\exp\{\int_{0}^{t}[\lambda (s)+\mu (s)]\;ds\}}. & \end{array}$$

If λ and μ are constant this simplifies to


(5.12) $$\begin{array}{ccc} & p_{1}(t)=\frac{e^{(\mu -\lambda )t}{\left(\lambda -\mu \right)}^{2}\rho }{[(\mu -[1-\rho ]\lambda )e^{(\mu -\lambda )t}-\lambda \rho {]}^{2}}. & \end{array}$$

In the case that ρ=1 this simplifies to


(5.13) $$\begin{array}{ccc} & p_{1}(t)=\frac{(\lambda -\mu {)}^{2}e^{(\lambda -\mu )t}}{[\lambda e^{(\lambda -\mu )t}-\mu {]}^{2}}, & \end{array}$$

which matches the result obtained by solving eq. 5.7 of [10].

## 6. Tree likelihoods under the generalized birth–death process and the generalized Yule process

Kubo & Iwasa [14] considered a GBDP in which λ(t) varies through time and μ is constant, and another GBDP in which μ(t) varies through time while λ is constant. They noted that both GBDPs produce the same likelihood on LTT data as a generalized Yule process (GYP) with a time-variable birth rate (and with μ=0). The fact that multiple GBDPs produce an identical PDF for a phylogeny means that the GBDPs are not statistically identifiable by the phylogeny (see definition in §7 below). For example, the model of variable birth rate λ(t) with a constant death rate μ and the model of variable birth rate λ(t) with zero death rate μ=0 are two distinct models with different parameter values that are not identifiable using LTT data.

Louca & Pennell [8] considered GBDPs with both variable birth rate λ(t) and variable death rate μ(t), and found that they produce the same likelihood on species divergence times as a GYP with a time-variable birth rate. Indeed there is a whole class of GBDPs with different time-dependent birth and death rates that have the same likelihood (are ‘congruent’ in the terminology of [8]) and are thus non-identifiable. Note that to demonstrate non-identifiability it is sufficient to show that any GBDP can be represented as a GYP. Suppose that the GBDP starts at time $t_{0}$ with a single lineage. The PDF of divergence times (i.e. the likelihood) under the GBDP is


(6.1) $$\begin{array}{ccc} & f(𝐭,n|t_{0})=p_{1}(t_{0})\prod_{i=1}^{n-1}[\lambda (t_{i})p_{1}(t_{i})], & \end{array}$$

where $p_{1}(t)$ is given in equation (5.7). This is the same as the likelihood under the GYP with a time-dependent birth rate


(6.2) $$\begin{array}{ccc} & \lambda_{p}(t)=\lambda (t)[1-p_{0}(t)]. & \end{array}$$

Here $\lambda_{p}(t)$ is called the ‘pulled’ speciation rate in [8] and is a function of λ(t), μ(t) and ρ in the original GBDP. Define


(6.3) $$\begin{array}{ccc} & \Lambda_{p}(t)=\int_{0}^{t}\lambda_{p}(s)\;ds. & \end{array}$$

Then the equivalent GYP likelihood is


(6.4) $$f_{p}(t,n|t_{0})=e^{-\Lambda_{p}(t_{0})}\prod_{i=1}^{n-1}\lambda_{p}(t_{i})e^{-\Lambda_{p}(t_{i})}=e^{-\int_{0}^{t_{0}}\lambda (s)[1-p_{0}(s)]\;ds}\prod_{i=1}^{n-1}\lambda (t_{i})[1-p_{0}(t_{i})]e^{-\int_{0}^{t_{i}}\lambda (s)[1-p_{0}(s)]\;ds}.$$

## 7. Identifiability of birth–death processes

If the probability distributions of the data X are identical under model m with parameters θ and under model $m^{\prime }$ with parameters $\theta^{\prime }$, with


(7.1) $$\begin{array}{ccc} & f(X|m,\theta )=f(X|m^{\prime },\theta^{\prime }) & \end{array}$$

for essentially all possible data X, the models are non-identifiable by data X. Both the terms non-identifiability and unidentifiability are used in the literature and should be considered synonyms. Note that the object of identifiability is the model or parameters, the object of statistical inference. One may use the term *within-model non-identifiability* if $m=m^{\prime }$ and $\theta \neq \theta^{\prime }$, or *cross-model non-identifiability* if $m\neq m^{\prime }$. In the former case, two sets of parameter values in the same model are non-identifiable, whereas in the latter two distinct models are non-identifiable. Non-identifiability reflects an essential indeterminancy in model or problem formulation [29, p. 54] and the definition concerns infinite data or all data (except for isolated data points that collectively have zero probability of occurrence, as implied in the term ‘essentially’ in equation (7.1).

Some authors [30] define *practical non-identifiability* as ‘the case when distinct parameter combinations cannot be told apart from the limited number of observations available in practice'. Thus, practical non-identifiability simply means low information content in practical datasets. We avoid such terminology. Also, identifiability depends on the type of data; a model that is non-identifiable with data X may be identifiable with extended data $X^{\prime }$. For example, models of between-species introgression are known to cause non-identifiability issues [31–33]. Introgression between sister lineages is non-identifiable using data of one sequence per species per locus but is identifiable when multiple samples are available per species [34,35]. Some models of gene flow are non-identifiable using gene tree topologies, but are identifiable using gene trees with branch lengths or sequence data [36].

Non-identifiability comes in many stripes (table 1). In some cases θ and $\theta^{\prime }$ under the same model represent isolated points in the parameter space. One of the best known such cases is the non-identifiability of the label-switching type, as occurs in Bayesian clustering. Let data $X=\{x_{i}\}$ be a sample from a mixture of two normal distributions, $\mathbb{N}(\mu_{1},\sigma_{1}^{2})$ and $\mathbb{N}(\mu_{2},\sigma_{2}^{2})$, with the mixing proportions $p_{1}$ and $p_{2}=1-p_{1}$. Let $\theta =(p_{1},\mu_{1},\sigma_{1}^{2},\mu_{2},\sigma_{2}^{2})$ be the parameter vector. Then $\theta^{\prime }=(p_{2},\mu_{2},\sigma_{2}^{2},\mu_{1},\sigma_{1}^{2})$ will have exactly the same likelihood, with $f(X|\theta )=f(X|\theta^{\prime })$ whatever data X are. Thus θ and $\theta^{\prime }$ are non-identifiable. In effect, the labels ‘group 1’ and ‘group 2’ are switched between θ and $\theta^{\prime }$. This non-identifiability is known as a *label-switching* problem. Models with label-switching non-identifiability can still be used for inference. For example, a *relabelling algorithm* can be used to post-process the MCMC sample to fix the label-switching issue [38,39]. Concerning the linear BDP, several authors have observed that the likelihood of the times under a BDP conditioned on a fixed number of lineages is invariant when the birth rate λ and the death rate μ are swapped [24,25]. This non-identifiability due to the symmetry in λ and μ is thus of the label-switching type, even though the two models are very different biologically. Similar non-identifiability (both within-model and cross-model) of the label-switching type occurs in models of bidirectional introgression in analysis of multilocus genomic data under the multispecies coalescent model [40].

**Table 1.** Summary of different types of non-identifiability discussed in this article. BDP, birth–death process; GBDP, generalized birth–death process.

condition	identifiability	type	references
*inference under BDPs*			
GBDP with continuous rate functions λ(t),μ(t)	non-identifiable	cross-model	[8]
GBDP with piecewise-constant rate functions λ(t),μ(t)	identifiable	cross-model	[27]
GBDP with piecewise-polynomial rate functions λ(t),μ(t)	identifiable	cross-model	[37]
BDP with constant rates λ,μ	θ=(λ,μ) and $\theta^{\prime }=(\mu ,\lambda )$ are non-identifiable	within-model, label-switching type	[24,25]
*other examples discussed in this article*			
Bayesian clustering	parameters for different clusters are non-identifiable	within-model, label-switching type	[38,39]
molecular clock dating without fossil calibrations	times and rate are non-identifiable	within-model, dimension reduction	[15]

Another common type of non-identifiability occurs when the likelihood function depends on the parameters θ only though a function h(θ); in other words, the likelihood L(θ) can be written as L(h(θ)), with h(θ) typically of a lower dimension than θ. Then θ and $\theta^{\prime }$ are non-identifiable if $h(\theta )=h(\theta^{\prime })$. Consider, for example, the estimation of divergence times $\text{𝐭}=(t_{1},\cdots \;,t_{5})$ of figure 1 in a molecular clock dating analysis using a sequence alignment. There are six parameters in the model, $\theta =(\mu ,t_{1},\cdots \;,t_{5})$, where μ is the mutation rate. However, the likelihood for the sequence data depends on $h(\theta )=(t_{1}\mu ,t_{2}\mu ,t_{3}\mu ,t_{4}\mu ,t_{5}\mu )$, which has five dimensions. If we multiply μ by a factor c and divide all times by the same factor, h(θ) remains unchanged and all parameters generated through this transform are non-identifiable. There is a one-dimensional ridge on the likelihood surface along which the likelihood is constant.

Non-identifiability of certain parameters may occur when some other parameters take particular values. This is known as *local non-identifiability*. For example, in the clustering problem, if $p_{2}=0$, then $\mu_{2}$ and $\sigma_{2}^{2}$ are non-identifiable. In the piecewise-constant model of variable speciation rate (§7b), if a time period has zero duration, the rate for that time period will be non-identifiable. Priors may be used to increase the separation between change points.

### (a) Non-identifiability of a generalized birth–death process

Here we illustrate the non-identifiability of the GBDP as discussed in [8,14] using a simple example. We show that the constant-rate BDP with complete sampling ρ=1 produces the same likelihood for LTT data as a pure-birth process with variable birth rate. As a result, the two models are non-identifiable.

Examining equations (6.1) and (6.4) we see that the two models are not identifiable if the following equality holds:


(7.2) $$\begin{array}{ccc} & \lambda p_{1}(t)=\lambda_{p}(t)e^{-\Lambda_{p}(t)}. & \end{array}$$

On one hand, substituting equation (5.13) into the left hand side of equation (7.2) gives


(7.3) $$\begin{array}{ccc} & \lambda p_{1}(t)=\frac{\lambda (\lambda -\mu {)}^{2}e^{(\lambda -\mu )t}}{{\left[\lambda e^{(\lambda -\mu )t}-\mu \right]}^{2}}. & \end{array}$$

On the other hand, from the constant rate BDP we have


(7.4) $$\begin{array}{ccc} & \Lambda_{p}(t)=\int_{0}^{t}\lambda [1-p_{0}(s)]\;ds=(\lambda -\mu )t+\log\left(\frac{\lambda -e^{(\mu -\lambda )t}\mu }{\lambda -\mu }\right), & \end{array}$$

and by substituting equation (5.6) into equation (6.2) we obtain


(7.5) $$\begin{array}{ccc} & \lambda_{p}(t)=\lambda [1-p_{0}(t)]=\lambda \left(1-\frac{\mu (e^{(\lambda -\mu )t}-1)}{\lambda e^{(\lambda -\mu )t}-\mu }\right). & \end{array}$$

Substituting equations (7.4) and (7.5) into the right hand side of equation (7.2) gives


(7.6) $$\begin{array}{ccc} & \lambda_{p}(t)e^{-\Lambda_{p}(t)}=\frac{\lambda (\lambda -\mu {)}^{2}e^{(\lambda -\mu )t}}{{\left[\mu -\lambda e^{(\lambda -\mu )t}\right]}^{2}}. & \end{array}$$

**Figure 2.**
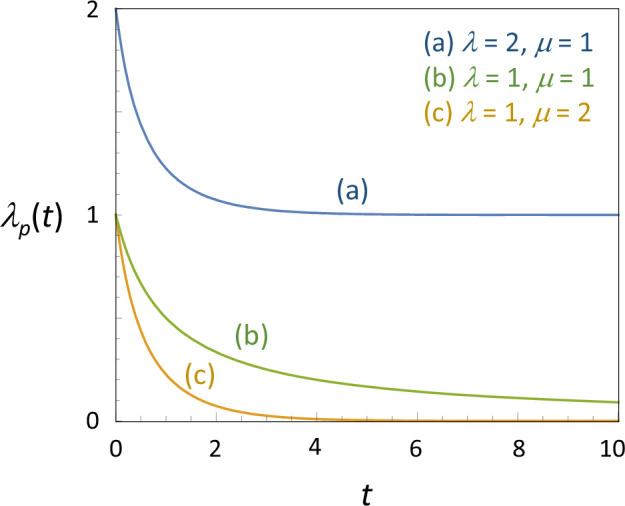
The ‘pulled speciation rate’, $\lambda_{p}(t)$, for the equivalent generalized Yule process of [8], which is non-identifiable with the birth–death process (BDP) model with constant birth and death rates (λ,μ) for three sets of parameter values. The BDP model with constant birth rate λ and constant death rate μ is non-identifiable, using lineage through time data, with the Yule process with variable birth rate $\lambda_{p}(t)$ given in equation (7.5).

Equation (7.3) and equation (7.5) are equal for any t,λ,μ. Thus, the likelihood under the constant-rate BDP (equation 6.1) and the likelihood under the pure-birth process with variable rate $\lambda_{p}(t)$ of equation (7.5) (equation (6.4)) are equal. Note that both models are special cases of the GBDP, so that those two GBDP models are non-identifiable. Figure 2 shows the relationship between $\lambda_{p}(t)$ of the GYP (equation (7.5)) and the constant rates λ and μ in the BDP.

### (b) Identifiability of birth–death processes with piecewise-constant rates

The GBDP with arbitrary speciation and extinction rate functions appears to be too general to allow identifiability. Legried & Terhorst [27] considered a restricted class of GBDPs with rate functions λ(t) and μ(t) to be piecewise-constant over K pieces (time periods). Such models are also known as *multiple-change-point models* in the statistics literature [41], with the boundaries between pieces of constant rate known as change points. Legried & Terhorst [27] showed that these models are identifiable as long as the number of tree tips is not too small relative to K. They further argue that with a large K these models can approximate any GBDP to an arbitrary degree, although this does not mean that real datasets contain information to recover arbitrary rate functions with any reliability (see below). Note that the result of §7a, that the BDP with constant rates λ and μ and the pure-birth process with variable rate $\lambda_{p}(t)$ are non-identifiable, is not in contradiction with Legried & Terhorst [27]. The variable-rate pure-birth process of equation (7.5) is not within the set of models considered by those authors, and there does not exist a piecewise-constant rate function for the pure-birth process that gives the same likelihood as the constant-rate BDP.

In simple cases (with certain regularity conditions [42] assumed), identifiability of parameters is indicated by the uniqueness of the maximum likelihood estimates and the non-singularity of Fisher’s information matrix, evaluated at the true parameter value [42,43]. Here we use this idea to illustrate that the simplest member of this class of cladogenesis models, a Yule process with two birth rates on different intervals, is identifiable. For simplicity, we assume that the time of the rate change is specified.

Consider a pure-birth process with complete sampling that begins at time $t_{0}$ in the past with rate $\lambda_{0}$ and switches to rate $\lambda_{1}$ at time T. With t>T assumed, equation (5.11) simplifies to


(7.7) $$\begin{array}{ccc} & p_{1}(t)=e^{-\int_{0}^{t}\lambda (s)\;ds}=e^{-[\lambda_{0}(t-T)+\lambda_{1}T]}=e^{-\lambda_{0}(t-T)}e^{-\lambda_{1}T},\;t>T. & \end{array}$$

This is also the probability that the lineage does not split over either time interval (t,T) or (T,0).

**Figure 3.**
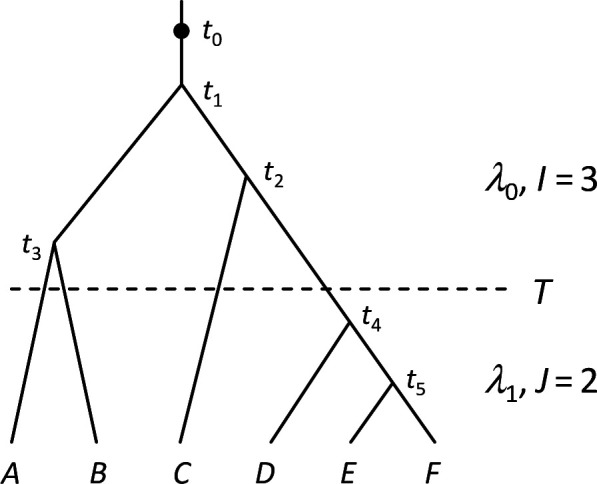
A piecewise-constant rate Yule model with two time periods of different rates. During the time period (0,T), the birth rate is $\lambda_{1}$ and J=2 speciation events are observed, whereas in the time period ($T,t_{0}$), the birth rate is $\lambda_{0}$ and I=3 speciation events are observed.


Now consider the reconstructed tree of n lineages where one lineage exists at time $t_{0}$, I lineages were born during interval $\left(t_{0},T\right)$ and J were born during (T,0) (figure 3). Let ${𝐭}^{0}$ and ${𝐭}^{1}$ be vectors of times of speciation events occurring in the epochs $\left(t_{0},T\right)$ and (T,0), respectively. For simplicity, we assume that $t_{0}$ and T are known, and there are only two parameters to estimate in the model: $\lambda_{0},\lambda_{1}$. The likelihood for the observed times, according to equation (4.6), is

(7.8) $$\begin{array}{rl} L(\lambda_{0},\lambda_{1}) & =f(t|t_{0},n)\\ & =\prod_{i=1}^{I}i\lambda_{0}e^{-\lambda_{0}(t_{i}^{0}-T)-\lambda_{1}T}\prod_{j=1}^{J}(I+j)\lambda_{1}e^{-\lambda_{1}t_{j}^{1}},\\ & =(I+J)!\lambda_{0}^{I}e^{TI(\lambda_{0}-\lambda_{1})}e^{-\lambda_{0}(\sum_{i=1}^{I}t_{i}^{0})}\lambda_{1}^{J}e^{-\lambda_{1}(\sum_{j=1}^{J}t_{j}^{1})}, \end{array}$$

with the log-likelihood:


(7.9) $$\begin{array}{ccc} & \log\;L=I\;\log\;\lambda_{0}+J\;\log\;\lambda_{1}+TI(\lambda_{0}-\lambda_{1})-\lambda_{0}\sum_{i=1}^{I}t_{i}^{0}-\lambda_{1}\sum_{j=1}^{J}t_{j}^{1}. & \end{array}$$

The partial derivatives are


(7.10) $$\begin{array}{ccc} & \frac{\partial \;\log\;L}{\partial \lambda_{0}}=\frac{I}{\lambda_{0}}+IT-\sum_{i=1}^{I}t_{i}^{0},\;\frac{\partial \;\log\;L}{\partial \lambda_{1}}=\frac{J}{\lambda_{1}}-IT-\sum_{j=1}^{I}t_{j}^{1}, & \end{array}$$

while the second derivatives are


(7.11) $$\begin{array}{ccc} & \frac{\partial^{2}\log\;L}{\partial \lambda_{0}^{2}}=-\frac{I}{\lambda_{0}^{2}},\;\frac{\partial^{2}\log\;L}{\partial \lambda_{1}^{2}}=-\frac{J}{\lambda_{1}^{2}}. & \end{array}$$

The maximum likelihood estimates are thus


(7.12) $${\hat{\lambda }}_{0}=\frac{I}{(\sum_{i=1}^{I}t_{i}^{0})-IT},\;{\hat{\lambda }}_{1}=\frac{J}{(\sum_{j=1}^{J}t_{j}^{1})+IT}.$$

The information matrix is


(7.13) $$\begin{array}{ccc} & F=-\left(\begin{array}{cc} \frac{\partial^{2}\log\;L}{\partial \lambda_{0}^{2}} & \frac{\partial^{2}\log\;L}{\partial \lambda_{0}\partial \lambda_{1}}\\ \frac{\partial^{2}\log\;L}{\partial \lambda_{1}\partial \lambda_{0}} & \frac{\partial^{2}\log\;L}{\partial \lambda_{1}^{2}} \end{array}\right)=\left(\begin{array}{cc} \frac{I}{\lambda_{0}^{2}} & 0\\ 0 & \frac{J}{\lambda_{1}^{2}} \end{array}\right). & \end{array}$$

The true parameter values are positive real numbers. If I>0,J>0, the information matrix will be non-singular and the parameters are identifiable. Note that here we have conditioned on the numbers of speciation times in each epoch: I,J. In the constant-rate Yule process described in §3, the number of times (n−1) is given by the number of tips n. Similarly, in the two-rate process n=I+J+1.

## 8. The limits of statistical information

The fact that the GBDP with piecewise-constant speciation and extinction rates is identifiable [27] may be encouraging for biologists interested in inferring speciation and extinction rates from reconstructed phylogenies. However, as noted by [27] identifiability does not ensure that information is available in real datasets to produce parameter estimates that are reliable and precise enough to draw useful biological conclusions. Simulations and other exploratory analyses may be used to determine how informative typical datasets are. We note that when the speciation rate and extinction rate vary over time, there is a general difficulty of small sample sizes. The case is similar to a coin-tossing experiment in which a sequence of coins with different probabilities for heads are flipped, each only once or a very few times, with the aim of estimating the probabilities for all coins. Furthermore, most studies applying the GBDP to reconstructed phylogenies (the LTT data) have assumed that the true divergence times are known, whereas in practice estimated divergence times involve considerable errors and uncertainties owing to the challenges of summarizing the fossil evidence, estimating branch lengths on molecular phylogenies, and the confounding effects of rates and times in analysis of molecular data (see [15] for a review).

Here we conduct a simulation experiment under a simple scenario with only one rate change to explore the information content in LTT data. We simulate divergence times with an extension of the two-rate Yule process model described in §7b above, and ask whether the change in speciation rate at time T can be recovered using a piecewise-constant Yule process model. We extend the model slightly by assuming that at the end of the first epoch (at time T), mass extinction occurs with the probability of any lineage surviving to be p while speciation rate is much higher after the mass extinction than before, with $\lambda_{1}\gg \lambda_{0}$. This mimics a mass-extinction scenario such as occurs in the fossil record at the Cretaceous–Palaeogene extinction event (K-Pg boundary), where extinction opens up new niches previously occupied by other species, increasing the speciation rate. However, many lineages existing before the extinction event will leave no descendants, so that it may not be possible to estimate the speciation rate for those lineages ($\lambda_{0}$) reliably.

For simplicity, we assume that the time of origin ($t_{0}$), the survival probability at extinction (p), the time of the mass extinction (T) and the numbers of speciation events in the two time epochs (I,J) are all known, so that there are only two parameters in the model: $\lambda_{0}$ and $\lambda_{1}$. If a lineage arises in the first epoch at time t>T the probability that it leaves exactly one descendant at present is given by averaging over the number of lineages (m) right before the mass extinction (with probability $p_{m}(t)$ of equation (3.7)), with only one of them surviving the mass extinction and leaving one descendant in the sample:


(8.1) $$p_{1}^{*}(t|\lambda_{0},\lambda_{1},T,p)=\sum_{m=1}^{\infty }e^{-m\lambda_{0}(t-T)}(e^{\lambda_{0}(t-T)}-1{)}^{m-1}\cdot mp(1-p{)}^{m-1}\cdot e^{-\lambda_{1}T}=\frac{pe^{\lambda_{0}(t+T)-\lambda_{1}T}}{[e^{\lambda_{0}T}(p-1)-e^{\lambda_{0}t}p{]}^{2}}.$$

Note that


(8.2) $$\begin{array}{ccc} & \lim_{p\to 1}\;p_{1}^{*}(t|\lambda_{0}=\lambda ,\lambda_{1}=\lambda ,T,p)=e^{-\lambda t}. & \end{array}$$

The likelihood is then


(8.3) $$\begin{array}{ccc} & L(\lambda_{0},\lambda_{1})=\prod_{i=1}^{I}\lambda_{0}ip_{1}^{*}(t_{i}^{0}|\lambda_{0},\lambda_{1},T,p)\prod_{j=1}^{J}\lambda_{1}(I+j)e^{-\lambda_{1}t_{j}^{1}}. & \end{array}$$

Note that the limit of equation (8.3) as p→1 is equation (7.8) as expected. The log-likelihood is


(8.4) $$\begin{array}{ll} \log L & =I\log\lambda_{0}+\sum_{i=1}^{I}\log[p_{1}^{*}(t_{i}^{0}|\lambda_{0},\lambda_{1},T,p)]+J\log\lambda_{1}-\lambda_{1}\sum_{j=1}^{J}t_{j}^{1},\\ & =I\log\lambda_{0}+\sum_{i=1}^{I}\log\left[\frac{pe^{\lambda_{0}(t_{i}^{0}+T)-\lambda_{1}T}}{[e^{\lambda_{0}T}(p-1)-e^{\lambda_{0}t_{i}^{0}}p{]}^{2}}\right]+J\log\lambda_{1}-\lambda_{1}\sum_{j=1}^{J}t_{j}^{1}. \end{array}$$

We simulated a dataset using $\lambda_{0}=0.025$, $\lambda_{1}=0.125$, $t_{0}=170$, T=66 and p=0.25. In this case, the process starts at 170 Ma with a speciation rate of 1 speciation per 40 Myr, and a mass extinction occurs at 66 Ma that caused 75% of species to become extinct (similar to the K-Pg extinction). Following this event, the speciation rate increases fivefold to 1.25 speciations per 10 Myr. The simulated data had I=3 and J=21 798, so the number of species in the reconstructed tree is 21 801, larger than many empirical datasets of this type.

**Figure 4.**
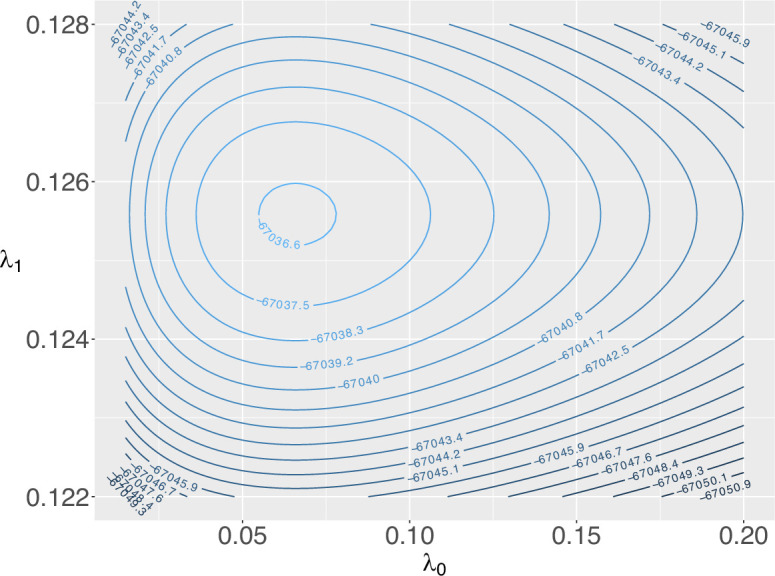
Log-likelihood surface for a realization of the two-rate epoch Yule process with a mass extinction event between epochs (figure 3). Time is given in millions of years and the true rates are $\lambda_{0}=0.025,\lambda_{1}=0.125$. LTT data are simulated assuming a mass extinction event at T=66 Ma, with the survival probability at the extinction to be p=0.25, and the process originated at $t_{0}=170$ Ma. Note the very different scales for the $\lambda_{0}$ and $\lambda_{1}$ axes.

A contour plot of the log-likelihood surface is shown in figure 4. Here we used the correct model with the log-likelihood given by equation (8.4), with p=0.25 and T=66 set to their true values. Although $\lambda_{1}$ is relatively precisely estimated there is a great deal of uncertainty about the speciation rate $\lambda_{0}$ before the mass extinction and it is difficult to infer a change in speciation rate after the extinction event from this dataset. Thus, even for very large datasets, it can be very challenging to identify even a large change in speciation rate between epochs. The two rates are identifiable in this case, but the information content in typical datasets may be very limited. Note that we have chosen this example simply to illustrate the potential effects of mass extinctions on rate inference; obviously a much more extensive simulation study would be needed to reach any general conclusions about the power of inference that is expected in real data analyses.

May *et al*. [44] developed a Bayesian inference model with both temporal shifts of speciation and extinction rates and periodic mass extinction events. Although they were specifically interested in detecting mass extinction events and estimating their time of occurrence (rather than rates before and after such events) they did find from simulations that the power to detect mass extinction events depends on how early they occur in relation to the phylogenetic tree. Ancient events near the root are more difficult to detect: ‘a mass-extinction event that occurs too close to the root of the study tree will be difficult to detect because too few lineages will have participated in that event’ [44, p.955]. This is similar to the situation concerning the ancestral rate ($\lambda_{0}$) in our example.

## 9. Discussion

Recent analyses suggest that the GBDP with arbitrary time-dependent speciation and extinction rates is not identifiable using LTT data, while the model becomes identifiable if the rate function is constrained to be piecewise-constant. We review those theoretical results, and discuss their implications to inference using empirical phylogenies. We demonstrate that in at least some cases even large phylogenies with thousands of species may not contain enough information to make useful inferences of past speciation and extinction rates.

Several recent papers have responded directly to the non-identifiability result of [8], even suggesting possible solutions. Morlon *et al*. [30] suggested the use of informative priors on rates or other sources of biological information to reduce the space of congruent models. Other authors [45] analysed empirical and simulated datasets to examine whether trends such as increasing rates of net cladogenesis can be identified, assuming that the congruent class of non-identifiable models has been found and relying on programs such as CRABS [46] to enumerate all such models. Extinction rates are particularly difficult to infer and one suggestion was to use a GYP to detect general trends of increasing or decreasing rates, again using the CRABS program [46] to identify congruent models of speciation and extinction rates. Kopperud *et al.* [45] found that many proposed congruent models were invalid (e.g. with negative extinction rates), so the space of valid models may be much smaller. They pointed out that statistical uncertainty about rates may be as important as unidentifiability of congruence classes.

The findings discussed in §7b that piecewise-constant [27] (and piecewise-polynomial, [37]) time-dependent rate functions are identifiable appear to offer hopes for evolutionary biologists interested in using timetrees to infer variable speciation and extinction rates. Although identifiability with infinite data is reassuring, the information in finite datasets about the rates during particular epochs may be very weak. To account for this, inference methods should be explicit about uncertainty of parameters and numbers of rate epochs. Bayesian methods with diffuse priors may prove more reliable than likelihood in reflecting the true levels of uncertainty in such cases. Another solution is to use simplified models that reduce the numbers of intervals with distinct rates, introduce autocorrelation, or reduce the number of parameters that need to be estimated from the data. Another venue for exploration is GBDP in which samples are collected through time (rather than all at the present time). Fossils add useful information and may make the model identifiable. A recent study [47] claims that the fossilized GBDP is identifiable.

Some readers may be surprised by the poor information content about the ancestral speciation rate before a mass extinction in our example in §8. However, in the simulation only four lineages existed prior to the extinction that are ancestral to all contemporary species, so there is clearly very limited information in this case. Some studies examining the power to infer ancient rate changes have produced more optimistic results. Stadler [48] developed a likelihood inference method for a BDP with piecewise-constant rates with a fixed number of rate shifts, attempting to infer both the number and time points of rate shifts and the rates on each interval. A small simulation with a single rate shift suggested that the method could accurately identify both the rates and the time of the rate shift. The simulations considered only very low extinction rates (up to one-quarter of the speciation rate in one simulation and three orders of magnitude lower extinction rates than the speciation rates in another). For mass extinction events one expects the extinction rate to be potentially orders of magnitude greater than the speciation rate, and a high extinction rate during a mass extinction will greatly reduce the number of ancestral lineages available for inferring rates from a reconstructed tree. Similarly, there is typically more power to detect recent than ancient diversification rate shifts (as found in [48] for mammalian diversification after the K-Pg mass extinction). More studies are needed to examine the performance of inference with extinction rates similar to or higher than speciation rates. The effect of mass extinctions in reducing information about past diversification rates shares many similarities with the related problem of inferring ancestral population sizes from genetic data in the presence of a population bottleneck [49,50]. In both cases, low numbers of lineages surviving the event lead to loss of information about rates prior to the event.

We have focused on the identifiability and inference properties of the GBDP with rate variation through time. Models of among-lineage rate variation, often referred to as *state-dependent extinction models* (SSEs) [13], are another generalization of the GBDP, so identifiability problems are likely to exist for such models as well. The difficulty of estimating parameters under SSE models has already been a subject of debate, although some of the problems have been due to implementations [7]. Existing inference methods invoking different assumptions are noted to produce very different results [51]. In some models, rates are positive random variables drawn from a continuous distribution at rate-change events, while in others there exist a fixed set of rates. Dragomir *et al.* [52] studied a multitype BDP (a particular type of SSE) with rates changing at random times but drawn from a fixed set of rates and proved that parameters under the model are identifiable. Recently, several contradictory studies have been posted that claim to prove either identifiability [53] or non-identifiability of general SSEs [54]. Hopefully, definitive answers will emerge soon. In conclusion, the problem of inferring rates of speciation and extinction using stochastic birth–death models, a legacy of Udny Yules’s [5] model in the 1920s, continues to provide rich challenges and unanswered questions for biologists and mathematicians in the twenty-first century.

## Data Availability

This article has no additional data.
